# Inhibiting ADAM17 enhances the efficacy of olaparib in ovarian cancer spheroids

**DOI:** 10.1038/s41598-024-78442-y

**Published:** 2024-11-06

**Authors:** Christoph Rogmans, Jan Dittrich, Emily Hamm, Jörg Paul Weimer, David Holthaus, Norbert Arnold, Inken Flörkemeier, Nicolai Maass, Peer Jansen, Astrid Dempfle, Dirk O. Bauerschlag, Nina Hedemann

**Affiliations:** 1 Department of Gynecology and Obstetrics, Kiel University and University Medical Center Schleswig-Holstein Campus Kiel, 24105 Kiel, Germany; 2 Laboratory of Infection Oncology, Institute of Clinical Molecular Biology, Kiel University and University Medical Center Schleswig-Holstein Campus Kiel, 24105 Kiel, Germany; 3 Institute of Medical Informatics and Statistics, Kiel University and University Medical Center, Schleswig-Holstein Campus Kiel, 24105 Kiel, Germany

**Keywords:** Ovarian cancer, PARP-inhibitor, Olaparib, Resistance, ADAM17, 3D tumor spheroids, Translational research, Experimental models of disease, Cancer therapeutic resistance, Gynaecological cancer

## Abstract

Acquired or de novo resistance to poly (ADP-ribose) polymerase inhibitors (PARPi) is a major challenge to ovarian cancer treatment. Therefore, strategies to overcome PARPi resistance are critical to improve prognosis. The purpose of this study is to evaluate whether inhibition of ADAM17 sensitizes ovarian cancer to treatment with olaparib, a PARPi, thereby bypassing resistance mechanisms and improving treatment response. Thus, we analyzed the effect of olaparib in combination with the ADAM17 inhibitor GW280264X in ovarian cancer using a 2D monolayer and a 3D spheroid model followed by a multicontent readout (viability, caspase activation and cytotoxicity). To emphasize the translational aspect of our work, we performed corresponding experiments on primary cells derived from ovarian cancer patients initially screened for their mutation status of the breast cancer gene (BRCA 1/2). In 2D, we observed a significant reduction in cell viability and a subsequent increase in apoptosis of the combined treatment (olaparib + GW280264X) compared with olaparib mono-treatment. The combined treatment allows a substantial dose reduction of olaparib rendering a strong synergistic effect. Using a 3D spheroid model from primary cells, we confirmed the 2D monoculture results and demonstrated not only increased caspase activity under the combined treatment but also a substantial gain in cytotoxicity compared to the mono-treatment. Our study proposes ADAM17 inhibition sensitizing ovarian cancer to olaparib treatment and improving treatment response.

## Introduction

Ovarian cancer (OvCa) has the highest mortality rate among gynecologic tumors^1^. In 2023, the American Cancer Society estimates around 19,710 initial diagnoses and over 13,200 deaths in the US alone^2^. As there are no early symptoms, ovarian cancer is diagnosed at an advanced stage of the disease in almost 75% of cases^3^. This is associated with limited treatment options and reduced overall survival. In addition, up to 80% of patents develop a recurrence within the first 2 years after diagnosis, 60% of patents even within the first 6 months^4^. One of the main reasons is the development of resistance to therapeutic agents, which results in tumor recurrence and metastasis.

High-grade serous ovarian cancer (HGSOC) represents the most common histologic subtype and has a high incidence of defects in homologous recombination (HRD), as well as mutations in *BRCA1/2*^5^. A germline or somatic mutation of *BRCA1/2* is known to be present in up to 26% of ovarian cancers^6^. Based on recent estimates, the cumulative risk of ovarian cancer development for *BRCA1* mutation carriers is 39% up o the age of 69, and 11–22% for *BRCA2* mutation carriers. According to the Cancer Genome Atlas, up to 25% of ovarian cancer patients also carry genetic alterations in other homologous recombination (HR) genes, such as *EMSY*,* PTEN*,* RAD51C*,* ATM/ATR*,* FANCM*, so HR deficiency is present in approximately 50% of GSOC^6,7^. For this, a corresponding score is calculated using the Myriad myChoice CDx test^8^.

Despite many efforts, there have been few innovations in the chemotherapy regime in recent decades. Staging surgery is followed by platinum-based chemotherapy with paclitaxel. However, the angiogenesis inhibitor bevacizumab, showed a significant prolongation of progression free survival (PFS) in the advanced tumor stages^9^. Another recent breakthrough is poly ADP ribose polymerase inhibitors (PARPi), which are approved in maintenance therapy after completion of platinum-based chemotherapy. They can be used with the highest efficacy in the presence of a germline mutation or a positive HRD score, given there has been a complete or at least partial response to platinum-based chemotherapy^10–12^. In addition, the AGO-OVAR-2.31-Study (OReO) demonstrated that patients who have progressed on a prior PARPi therapy could benefit from re-challenge with PARPi after platinum response and had significantly longer progression-free survival (PFS 5.3 month olaparib vs. 2.8 month placebo)^13,14^. The Evolve study revealed that revision mutations in *BRCA1/BRCA2* and *RAD41B*, as well as increased expression of ABCB1, lead to rapid development of resistance and are associated with poor outcome^15^.

Tumors have various repair mechanisms, such as homologous recombination or base excision repair, to correct DNA damage. If HRD or *BRCA* mutations are present, the sensitivity to DNA-damaging therapy is increased^16^. Poly (ADP-ribose) polymerase (PARP) is essential in the repair of single-strand DNA breaks. PARPi bind to the active site of PARPs associated with DNA, preventing its dissociation (DNA-trapping). DNA single-strand breaks accumulate, which lead to double-strand breaks in DNA replication. This induces chromosomal instability, which triggers apoptosis, particularly in *BRCA1/2* deficient cells. PARPi have played a major role in the therapy of ovarian cancer for several years^17^. However, analogous to chemotherapeutic agents, such as carboplatin and paclitaxel, this therapy can also exhibit a rapid development of resistance, leading to therapy limitation. Resistance to both PARPi and platinum-based chemotherapy is based on common resistance mechanisms. Therefore, resistance to platinum-based chemotherapy is a strong predictor of PARPi resistance.

A disintegrin and metalloproteases 17 (ADAM17) is overexpressed in many tumors such as lung, breast and ovarian cancer and plays a crucial role in tumor proliferation, progression and migration^18–20^. Ectodomain shedding releases over 80 different substrates that activate intracellular signaling cascades such as the EGFR/PI3K/AKT pathway or Notch signaling^21,22^. The release of activators of the EGF receptor, such as amphiregulin, HBEGF, TGFα, which trigger proliferation and suppress apoptosis, has been studied most extensively^23,24^. Because ADAM17 is upstream of the EGFR, PI3K/AKT and MAPK signaling pathways, which are key players in chemoresistance, Kyula et al. were able to link ADAM17 to various resistance mechanisms^25^.

We recently demonstrated that ADAM17 plays a critical role in the development of cisplatin resistance^26,27^. Inhibition of ADAM17 induced sensitization of tumor cells to cisplatin-induced apoptosis, which was accompanied by a significant reduction in cell viability. These results suggest that inhibition of ADAM17 may reduce the development of secondary resistance mechanisms. This approach will now be applied to PARPi, which, in addition to chemotherapeutic agents, is playing the essential role in maintenance therapy of ovarian cancer. Therefore, we investigated whether inhibition of ADAM17 can lead to sensitization of cells to olaparib treatment, the most commonly used PARPi in clinical practice. The overall goal was to validate potential new targets for combination therapies in ovarian cancer and thus circumvent resistance mechanisms. To improve the validity of the results, we obtained primary cells from ovarian cancer patients and performed the experiments in a 3D tumor spheroid model that represents the in vivo conditions, with different cell layers, different diffusion properties and cell-cell interactions more realistic.

## Results

### Characterization of OvCa cell lines and primary cells: primary cells harbor less mutations in the risk genes compared to established cell lines

Since clinically the response to treatment with a PARPi depends significantly on the respective HRD score, the HRD score was determined using aCGH for the cell lines examined. Based on preliminary analyses we selected three cell lines, one with high, one with intermediate, and one with low HRD score for further investigation. OVCAR-8 exhibited a high HRD score of 69, SKOV-3 an intermediate HRD score of 31 and IGROV-1 a low HRD score of 4 (Table 1). In addition to the determination of the HRD score, an analysis of all known risk genes for ovarian cancer was performed using the TruRisk Genepanel assembled by the German Consortium for hereditary Breast and Ovarian Cancer (Table 2). In particular, IGROV-1 reveals a homozygous mutation of the known missmatch repair genes MSH6 and MLH1. In addition, heterozygous mutations of the tumor suppressor genes *BRCA1* and *BRCA2* are present. It is interesting to note that there are also multiple mutations in genes regulating the PI3K and MAP3KI pathways. Despite the low HRD score, gene analysis in IGROV-1 suggests a hypermutation of the cell line. The OVCAR-8 and SKOV-3 cell lines carried a homozygous mutation for the tumor suppressor gene TP53. In accordance with the clinical procedure, a TruRisk Genepanel analysis was first carried out on the primary cells. Only if the test result was inconspicuous the HRD score was also defined. Examination of the primary cells revealed that UF-364 and UF-510 also had a heterozygous *BRCA-1* mutation. The primary cells UF-357, UF-477 and UF-510 showed heterozygous *BRCA2* mutations. UF-352 carries two mutations of unclear significance in the ATM gene. However, these are currently not considered pathological. The HRD status of UF-352 was therefore additionally determined for further analysis. With an HRD score of 92, this indicates sensitivity to PARPi therapy. In general, fewer mutations of the risk genes are present in primary cells, compared to the established cell lines.

**Table 1:** Listing of the HRD score for the individual cell lines and primary cells.

Cells	LOH	TAI	LST	HRD-score
OVCAR-8	3	13	53	69
SKOV-3	10	9	12	31
IGROV-1	2	2	0	4
UF-352	18	11	63	92

The HRD score is calculated by summing the loss of heterozygosity (LOH), telomeric allelic imbalance (TAI) and large-scale state transitions (LST).

**Table 2:** Listing of pathogenic and likely pathogenic mutations of the individual cell lines and primary cells.

Cells		Chromosomes		HGVS	Protein	Gene	Frequency	Zygosity
IGROV-1		2		c.3254delC	p.(Thr1085Thrfs*5)	MSH6	95.07%	homoz.
		3		c.1344delG	p.(Glu448Glufs*43)	MLH1	48.88%	heteroz.
		3		c.1513delA	p.(Ser505Valfs*3)	MLH1	98.28%	homoz.
		3		c.112 C > T	p.(Arg38Cys)	PIK3CA	51.40%	heteroz.
		5		c.2157delA	p.(Leu719Leufs*17)	RAD50	48.70%	heteroz.
		5		c.862 C > T	p.(Arg288*)	MAP3KI	47,8%	heteroz.
		10		c.464 A > G	p.(Tyr155Cys)	PTEN	49.68%	heteroz.
		10		c.950_953delTACT	p.(Val317Valfs*3)	PTEN	45.96%	heteroz.
		13		c.18_19delAG	p.(Lys6Lysfs*7)	BRCA2	48.08%	heteroz.
		17		c.1961delA	p.(Lys654Serfs*47)	BRCA1	43.80%	heteroz.
OVCAR-8		17		c.376-1G > A		TP53	99.86%	homoz.
SKOV-3		3		c.3140 A > G	p.(His1047Arg)	PIK3CA	48.22%	heteroz.
		11		c.4237–2 A > G		ATM	50.87%	heteroz.
		17		c.267delC	p.(Pro89Profs*34)	TP53	88.64%	homoz.
UF-352		11		-	-	-	-	-
UF-357		13		c.7488_7492delinsAAGG	p.(Lys2497Argfs*27)	BRCA 2	50.00%	heteroz.
UF-364		17		c.3640G > T	p.(Glu1214*)	BRCA 1	50.00%	heteroz.
UF-477		13		c.5946del	p.(Ser1982Argfs*22)	BRCA 2	50.00%	heteroz.
UF-510		17		c.5179 A > T	p.(Lys1727Ter)	BRCA 1	50.00%	heteroz.

Analysis was performed using TruRisk gene panel. UF-352: no pathogenic or likely pathogenic mutation has been identified. HGVS: human genome variant nomenclature.

### Combination of olaparib and ADAM17 inhibition reduces cell viability and induces apoptosis in 2D monolayers

To investigate the potential combinatorial effects of ADAM10 (GI254023X)/ ADAM17 (GW280264X) inhibition and olaparib, viability and apoptosis was measured in cell lines with different HRD scores. Therefore, we treated IGROV-1, OVCAR-8 and SKOV-3 with 3 μm GI254023X (GI)/ GW280264X (GW)/ DMSO followed by olaparib after 24 h. Multiplex analysis to determine viability and apoptosis was performed after 72 h of incubation.

**Fig. 1:**
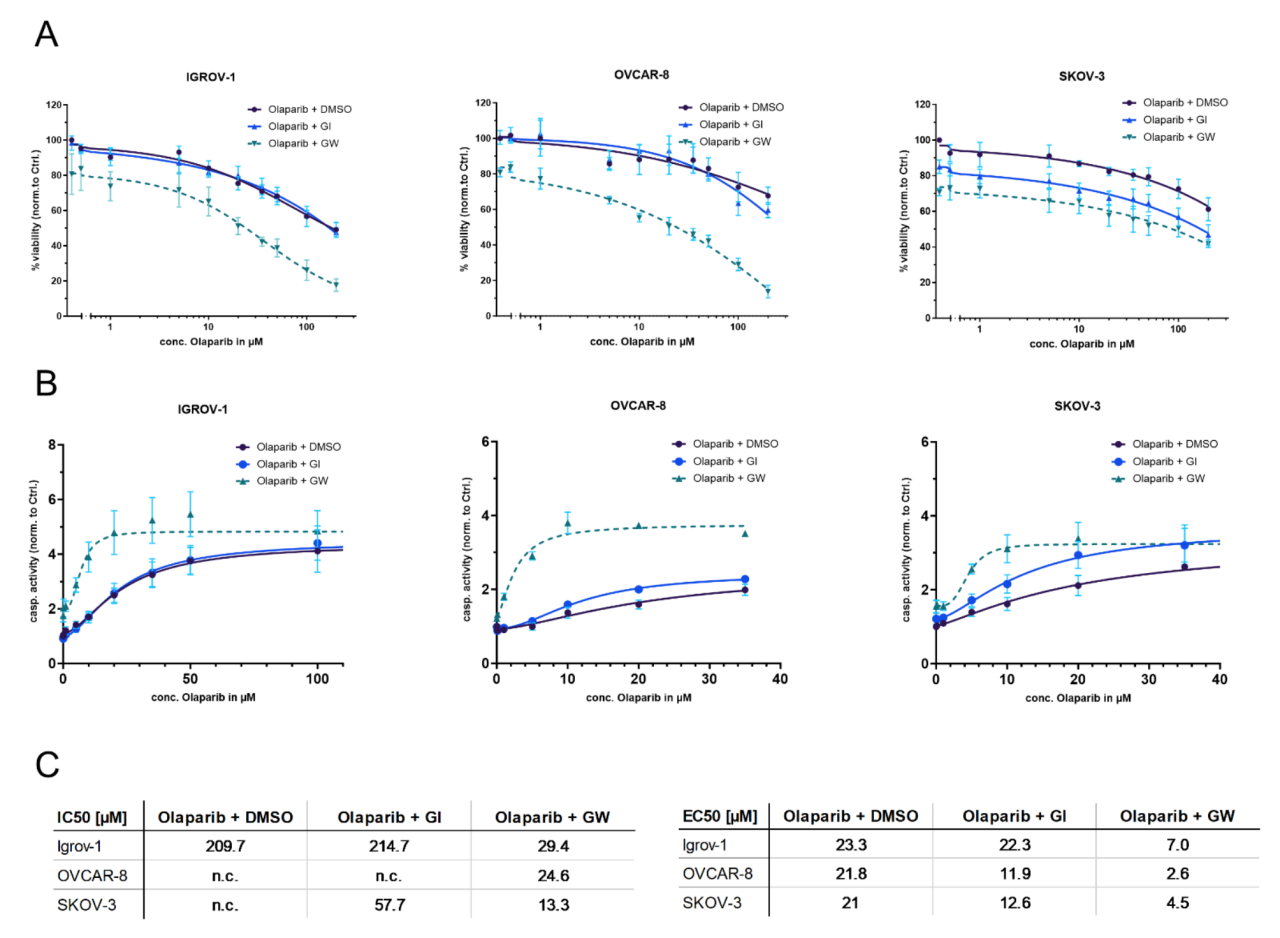
Inhibition of ADAM17 in combination with olaparib leads to a significant reduction in cell viability and a subsequent increase in apoptosis in all ovarian cancer cell lines. Ovarian cancer cell lines IGROV-1, OVCAR-8 and SKOV-3 were seeded in 2D monolayers and treated after 24 h with increasing concentrations of olaparib and a constant concentration of 3 µM GW, GI or DMSO. After 48 h, viability and caspase activity were determined using the ApoLive-Glo Mutliplex assay. (**A**) The combination treatment of olaparib and GW induces a significantly greater decrease in viability compared to mono-treatment with olaparib. (**B**) Even at low concentrations, the combination treatment (GW + olaparib) leads to a strong increase in apoptosis. (**C**) The IC_50_ and EC_50_ concentrations were calculated for each treatment. Data represent at least three biological replicates, indicated as Means with SEM. n.c.: non computable values.

Combined treatment with GW and olaparib showed a strong synergistic effect compared with olaparib monotherapy (Fig. 1A). In all investigated cell lines, there was a significant reduction in IC_50_ in the combinatorial treatment compared to olaparib monotherapy (*p* < 0.0001) (Fig. 1C). In contrast, inhibition of ADAM10 with GI in combination with olaparib showed significantly lower IC_50_ only in SKOV-3 (*p* < 0.0001), with no significant differences in IGROV-1 and OVCAR-8 (*p* = 0.90, *p* = 0.34). To quantify the dose reduction effect of the combination treatment, the DRI_50_ was calculated. The DRI_50_ represents the magnitude of dose reduction required to achieve IC_50_ compared to single treatment with olaparib. The DRI_*50*_ allows to distinguish between antagonistic (DRI_*50*_ > 1), additive (DRI_*50*_ = 1) and synergistic effects (DRI_*50*_ < 1) of the combinatorial treatment. Based on the assessed DRI, the additional inhibition of ADAM17 to olaparib treatment showed a strong synergistic effect with a dose reduction of olaparib to 2% in OVCAR-8 and SKOV-3, respectively, and 14% in IGROV-1 compared to monotherapy (Table 3). This effect was less pronounced with ADAM10 inhibition, resulting in a mild synergistic effect with OVCAR-8 (DRI 73%) and an enhanced effect with SKOV-3 (DRI 11%). No effect was indicated for IGROV-1. Congruent with these results, direct comparison between the two combination treatments (GW + olaparib vs. GI + olaparib) showed a more noticeable effect of ADAM17 inhibition (DRI_50_: 21–56%).

In addition to viability, we also analyzed caspase activity to calculate the half-maximal effective concentration (EC_50_) (Fig. 1B). Overall, the above results could be confirmed: The additional treatment with GW leads to an enhanced induction of apoptosis compared to treatment with olaparib only (Fig. 1B). The half-maximal effect could be achieved at 4–15% of the initial dose. Combination treatment of olaparib and GI again showed a minor dose reduction to 40–95%. Across all cell lines, a direct comparison of combination treatments between GI and GW calculated a dose reduction of 19–45% in favor of GW (Table 3). In summary, our results underline that inhibition of ADAM17 in combination with olaparib leads to a significant reduction in cell viability and a subsequent increase in apoptosis in all ovarian cancer cell lines. In addition, combination treatment allows a significant dose reduction of olaparib compared to mono-treatment.

**Table 3:** Dose reduction index at 50% effectiveness for the combinations of olaparib + GW, GI or DMSO.

DRI_50_ of IC_50_	Ola + DMSO vs. Ola + GI	Ola + GI vs. Ola + GW		Ola + DMSO vs. Ola + GW		*p*	
Igrov-1	1.02	0.35		0.14		***	
OVCAR-8	0.73	0.21		0.02		***	
SKOV-3	0.11	0.56		0.02		***	

DRI_50_ of EC_50_	Ola + DMSO vs. Ola + GI		Ola + GI vs. Ola + GW		Ola + DMSO vs. Ola + GW		*p*	
Igrov-1	0.95		0.31		0.15		***	
OVCAR-8	0.56		0.19		0.04		***	
SKOV-3	0.40		0.45		0.13		***	

The DRI allows to distinguish between antagonistic (DRI_50_ > 1), additive (DRI_50_ = 1) and synergistic effects (DRI_50_ < 1) of the combinatorial treatment. DRI_50_ were calculated by fitting standard four-parameter logistic regression dose-response assuming a common maximum (top), minimum (bottom) and slope parameter to compare IC_50_ and EC_50_ values of mono-treatment vs. combination treatment (GW + olaparib), respectively. Inhibition of ADAM17 to olaparib treatment vs. Olaparib mono-treatment revealed a highly significant reduction in DRI_50_ in all invastigated cell lines. *** *p* < 0.001.

### Combination treatment of ADAM17 inhibition and olaparib results in potent synergistic effects in primary ovarian cancer cells

**Fig. 2:**
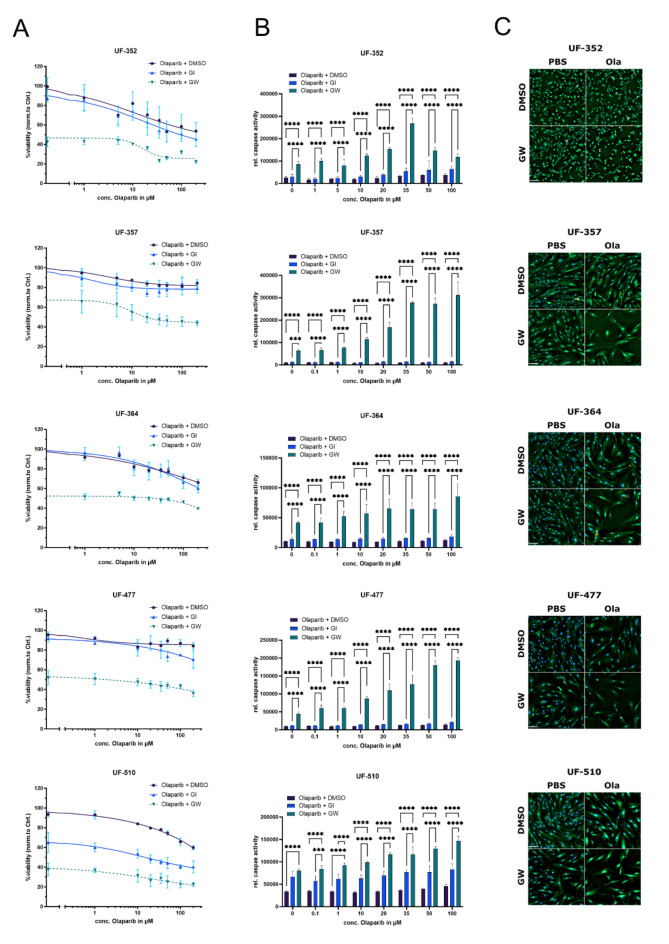
Combined treatment of ADAM17 inhibition and olaparib leads to strong synergistic effects in primary ovarian cancer cells. Primary cells were seeded in 2D monolayers and treated with increasing concentrations of olaparib and 3 µM GI, GW or DMSO. After 48 h, ApoLive-Glo Mutliplex assay was performed. Viability and caspase were normalized to control. (**A**) Mono-treatment with the ADAM17 inhibitor GW leads to a strong decrease in viability, which is further enhanced by the addition of olaparib. Cell viability was measured as relative fluorecence units (RFU). (**B**) Even low concentrations of olaparib in combination with GW revealed strong synergistic effects. Caspase 3/7 activity was quantified as relative luminiscence units. Data displayed as Mean ± SD. ***p = 0.001; ****p < 0.0001. (**C**) Triple staining of primary cells (propidium iodide (PI), Hoechst33342 and Calcein-AM) Staining corresponds to green: living cells, red: dead cells, blue: nuclei. The combination treatment shows a decrease in cell confluence and an enhanced PI signal. Ola = 10 µM olaparib. Scalebar: 200 μm.

In order to translate the findings to primary material, cells were isolated from tumor tissue of ovarian cancer patients and cultured for the following experiments. In addition, to clinical parameters (Table 4), TruRisk Genepanel analyses was performed for each patient (Table 2). Interestingly, in primary cells, mono-treatment with the ADAM17 inhibitor GW already induced significant cell death. GW reduced viability by over 40% in UF-352 (*p* = 0.0001), UF-364 (*p* = 0.0001) and UF-477 (*p* = 0.0001), respectively, and by over 50% in UF-510 (*p* = 0.0001) compared to untreated cells (Fig. 2A). With the addition of 20 µM olaparib, the viability of cells treated with the combination treatment further decreased by 50% (UF-352), 42% (UF-357), 39% (UF-364), 48% (UF-477), and even 68% (UF-510) compared to olaparib mono-treatment. In contrast, inhibition of ADAM10 did not lead to a significant reduction in viability (UF-352: *p* = 0.33; UF-357: *p* = 0.43; UF-364: *p* = 0.51; UF-477: *p* = 0.29) except for UF-510 (*p* = 0.0001). Relative caspase activity showed a similar effect: mono-treatment with GW demonstrated a significant increase in apoptosis in all primary cells (Fig. 2B). Inhibition of ADAM10 only showed a significant increase in apoptosis in UF-510. Under additional treatment with higher concentrations of olaparib, viability further decreased and induction of apoptosis increased strongly. Particularly focusing on the caspase results of UF-477 it is evident that olaparib alone only induces cell death to 1.3-fold even at high doses, while the addition of GW leads to a significant increase of more than 4-fold compared to baseline activity (Fig. 2B). Moreover, a direct comparison of the relative caspase activity at a concentration of 35 µM between the olaparib mono-treatment and the combinational treatment with GW shows a significant increase of 3-fold for UF-510, 6-fold for UF-364, 8-fold for UF-352, 10-fold for UF-477 and more than 26-fold for UF-357. Even at the lowest investigated olaparib concentrations, viability with olaparib + GW was much lower than with olaparib + DMSO, so that no sensible IC_50_ (or EC_50_) ratio and thus no DRI was estimated. To validate the results, in addition to the ApoLive-Glo Mutliplex assay, we visualized the therapeutic effect of olaparib and GW, GI and DMSO by live-dead and nucleus staining (Fig. 2C). In all five primary cells, the decrease in confluence with reduced Calcein-AM staining (living cells) and an increase in PI signal (dead cells) was observed not only in the mono-treatment with GW, but especially in the combination treatment. Taken together these results indicate that the combined treatment effect of GW and olaparib can also be transferred to primary cells.

**Table 4:** Clinical parameters of the examined patients.

Patients	Subtype	FIGO stage	Age at initial diagnosis	Resection of tumor
UF-352	HGSC	IIIc	59	R0
UF-357	HGSC	IIIc	52	R0
UF-364	HGSC	IIIc	49	R1
UF-477	HGSC	IIIc	59	R0
UF-511	HGSC	IIIc	56	R0

Shown in the table: histologic subtype, FIGO stage, age of diagnosis, resection of tumor mass under surgery. Abbreviations: HGSC: high grade serous ovarian cancer, FIGO: Fédération internationale de Gynécologie et d’Obstétrique, R0: tumor was completely removed, R1: tumor mass could not be completely resected.

### ADAM17 inhibition leads to increased cytotoxic effect under olaprib therapy in tumor spheroids

**Fig. 3:**
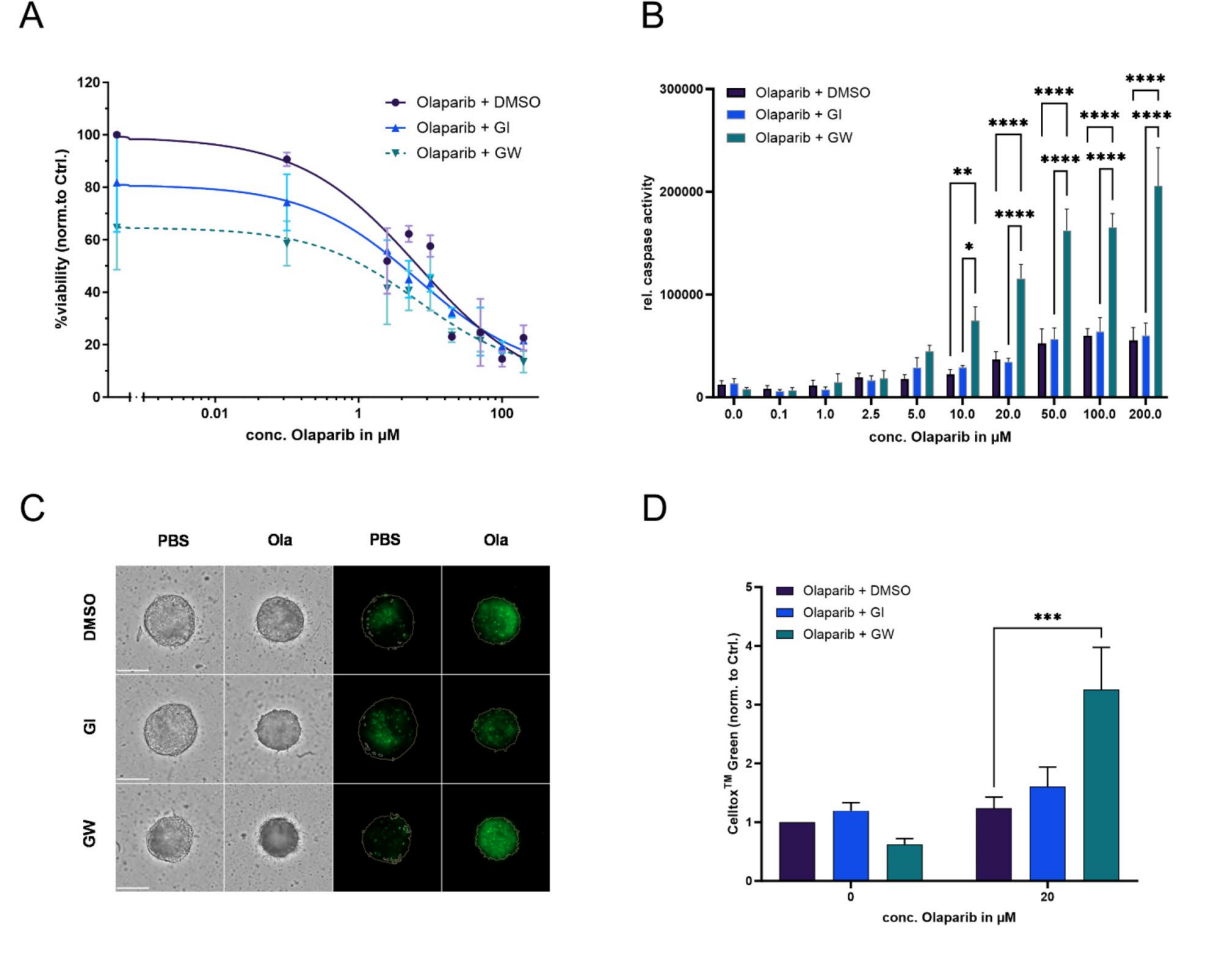
ADAM17 inhibition leads to increased cytotoxic effect under olaparib therapy in OVCAR-8 tumor spheroids. (A) Reduction of viability 72 h after treatment. Viability measured by RealTime-Glo MT Cell Viability assay. Data represented as Mean ± SEM. (B) Increase in caspase 3/7 activity normalized to the number of viable cells. Data displayed as Mean ± SEM. *p < 0.05, **p < 0.01, ****p < 0.0001. (C) CellTox Green staining confirms the enhanced effect of olaparib + GW. C: Representative brightfield images and CellTox Green staining. (D) Quantitative analysis of the corresponding CellTox Green fluorescence at PBS/ 20 µM olaparib. ***p < 0.001. Scalebar: 300 μm.

To strengthen the potent synergistic effect of combination treatment in 2D, we established a 3D model of tumor spheroids^27^. Pre-test showed that especially the cell line OVCAR-8 forms dense spheroids with a necrotic core. OVCAR-8 spheroids were treated with increasing concentrations of olaparib, as well as 3 µM GW, GI or DMSO, likewise to 2D models. Mono-treatment to inhibit ADAM17 revealed a 35% rduction in viability compared to control, whereas inhibition of ADAM10 has a minor effect (Fig. 3A). The calculated IC_50_ was significantly lower for olaparib + GW (2.90 µM), compared with olaparib + DMSO (9.77 µM), and olaparib + GI (6.60 µM) treatment, respectively. Consistent with this, the DRI_50_ pointed to a 69% dose reduction with the addition of ADAM17 inhibition to olaparib mono-treatment. Inhibition of ADAM10 resulted in a dose reduction of only 34%. oncurrently, from a dose of 10 µM olaparib, there is a significant increase in rel. caspase activity in olaparib + GW, compared to olaparib + DMSO, or olaparib + GI, indicating a strong synergistic effect (Fig. 3B). Inhibition of ADAM10 did not lead to an increased induction of apoptosis compared to olaparib mono-treatment. In addition, to validate and visualize the effect, we performed the CellTox Green Cytotoxicity Assay, allowing the detection of cytotoxic effect on cultured cells (Fig. 3C). In line with the 2D experiments, a strong increase in cytotoxicity was shown by ADAM17 inhibition, highlighting the synergistic effect. Quantitative evaluation of CellTox Green fluorescence demonstrates a 2.6-fold increase in apoptosis (*p* = 0.001) by ADAM17 inhibition compared to control, but no significant difference by inhibition of ADAM10 (*p* = 0.66) (Fig. 3D). Furthermore, representative brightfield images show not only a decrease in spheroid sizes under mono- treatments with GW or olaparib, but also morphological changes including disintegration of intercellular contacts of spheroids treated with both therapeutics. (Fig. 3C, lower right). In conclusion, the 2D cell culture results can also be demonstrated in a more complex model of ovarian cancer tumor spheroids: The effect of olaparib treatment is significantly enhanced by the additional inhibition of ADAM17, allowing a substantial dose reduction.

### The combinatorial effect of olaparib and GW can also be demonstrated in a translational approach on primary ovarian cancer spheroids

**Fig. 4:**
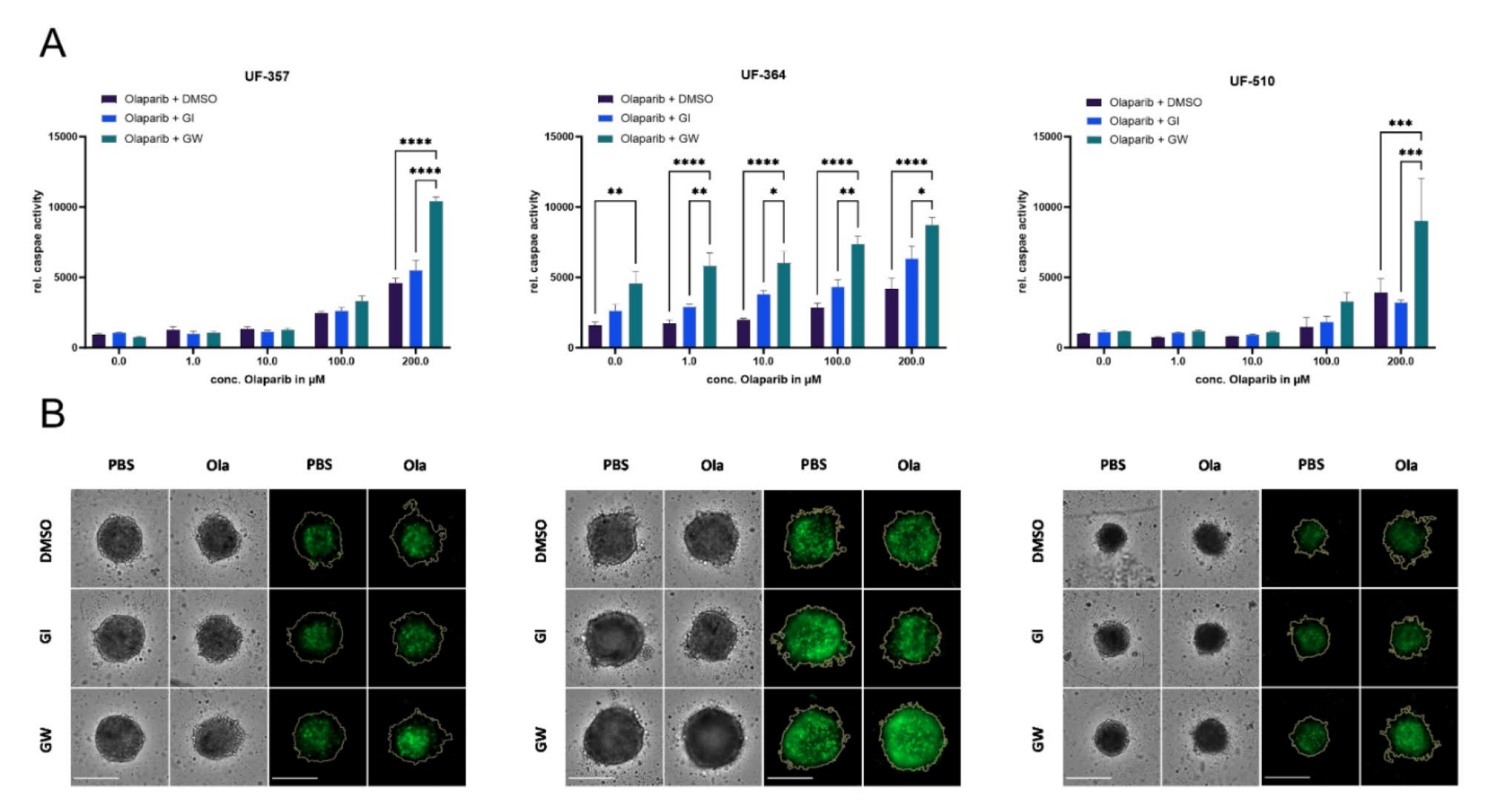
Combinatorial treatment effect of olaparib and GW in a translational approach using primary OvCa spheroids. Primary cells from two BRCA 1 (UF-364; UF-510) and one BRCA 2 mutated patient (UF-357) were grown as spheroids in ULA plates and analyzed for their response to olaparib and additional treatment either GI or GW. The solvent DMSO has been used as a control. (A) Relative (rel.) caspase activity was increased following treatment with olaparib and GW compared to olaparib mono-treatment. Data is presented as mean ± SD. *p < 0.05, **p < 0.01, ***p < 0.001, ****p < 0.0001. (B) Representative Brightfield (left) and CellTox Green (right) images of UF-357; UF-364 and UF-510 following the indicated treatment conditions, which generated the strongest combinatorial effect are displayed (UF-357: 0/100 µM olaparib; UF-364: 0/10 µM olaparib; UF-510: 0/200 µM olaparib). Scalebar: 250 μm.

In a second translational approach, we investigated drug response on 3D spheroids composed of primary ovarian cancer cells. Since primary cells can only be cultured for a limited number of passage cycles, the amount of cells for the following experiments was restricted. Accordingly, we limited ourselves with respect to the number of treatment concentrations. The primary spheroids of UF-364 already showed a 2.8-fold increase in caspase activity upon mono-treatment with GW (Fig. 4A), whereas GW mono-treatment had no effect in UF-357 and UF-510 cells. Moreover, small amounts of olaparib (1 µM -10 µM), which were not sufficient to increase caspase activity as mono-treatment, supplemented the effect of GW, resulting in a more than three fold increase in caspase activity compared to olaparib alone. In contrast, UF-357 and UF-510 cells, which neither responded to GW mono-treatment nor to low concentrations of olaparib (up to 10 µM) a combined effect was only detectable using significantly higher (100 µM and 200 µM) olaparib concentrations. UF-357 and UF-510 demonstrated a 2.2-fold and 2.3-fold increase in caspase activity, respectively, at an olaparib concentration of 200 µM. These results indicate that an initial cytotoxic effect induced by olaparib is required to enhance the therapeutic response by ADAM17 inhibition. In general, inhibition of ADAM10 only led to a slight induction of apoptosis in UF-364 primary spheroids. Representative bright field images show spheroid morphology following the different combinatorial treatment conditions. I.e. combination of GW and olaparib indicates loss of membrane integrity of the outer layer of the spheroidal cell cluster indicating strong combinatorial effects. Using CellTox Green staining as a marker for cytotoxicity, confirmed the combinatorial effect of olaparib in combination with ADAM17 inhibition (Fig. 4A, B). Overall, this translational approach suggests that ADAM17 enhances the effect of olaparib, particularly in BRCA mutated patients.

## Discussion

Despite the development of innovative therapeutic strategies, the 5-year survival rate for ovarian cancer is significantly lower than for the majority of gynecologic carcinomas. Often, the diagnosis is made at an advanced stage of the disease and the emergence of secondary resistance mechanisms to platinum-based chemotherapy and PARPi severely limit therapeutic options. In this study, we demonstrated that inhibition of ADAM17 significantly improved the treatment response of olaparib in ovarian cancer.

Reasons for the development of resistance are manifold. Interestingly, resistance to PARPi and platinum-based chemotherapy is based on common resistance mechanisms. Because resistance to platinum-based chemotherapy is a strong predictor of PARPi resistance, sensitivity to chemotherapy is a requirement for clinical application of PARPi. Several factors underlying these mechanisms have been identified: First, increased activity of drug efflux transporters has been observed, which decreases the concentration of PARPi intracellularly and reduces the therapeutic effect^28^. In a mouse model with *BRCA1*-deficient breast carcinomas, an association between PARPi resistance and overexpression of drug efflux transporter genes have been shown^29^. Another key mechanism of acquired platinum and PARPi resistance is the restoration of homologous recombination. In 46% of the platinum-resistant *BRCA* mutated high-grade serous ovarian carcinomas, reversion mutations in the *BRCA1/2* genes could be detected. *BRCA* mutations are often based on deletions that lead to a frameshift and subsequently reduce the functionality of the protein^30,31^. Due to high selection pressure during treatment, secondary mutations can be triggered, which restore the open reading frame and lead to reactivation of the BRCA protein activity. Overall, revision mutations are responsible for 20–25% of PARPi resstances in *BRCA* mutant patients^32^. Furthermore, HR deficiency can be caused by *BRCA* gene promoter methylation, which occurs in 15–20% of high-grade ovrian carcinomas^33^. Nesic et al. were able to show that heterozygous RAD51C methylation can confer acquired resistance^33^. Loss of *BRCA1* promoter methylation during PARPi therapy has been shown to contribute to reactivation of HR^34^.

Several approaches to overcome acquired PARPi resistance have been initiated: It was demonstrated that inhibition of PI3K-signaling leads to induced apoptosis by downregulation of BRCA 1/2 proteins^35,36^. This drug-induced “BRCAness” confers increased tumor sensitivity to PARPi^36^. Furthermore, inhibition of the RAS/RAF/MEK/ERK pathway has also been shown to reverse acquired PARPi resistance. Sun et al. found evidence that mutations in KRAS have an impact on PARPi resistance and are associated with higher homologous recombination capacity^37^. Accordingly, inhibition of MEK/ERK reduces several components of homologous recombination, and strong synergistic effects are reported in combination with PARPi^37^. Overall, both in vitro and in vivo data suggest that MEK/ERK inhibitors can increase PARPi-induced apoptosis and reverse acquired PARPi resistance.

The overarching challenge of kinase inhibition is the activation of bypass signaling pathways that evade the initial therapeutic effect and promote secondary drug resistance. Inhibition of a specific signaling pathway, such as the MAPK-signal pathway, reduces extracellular shedding of receptor tyrosine kinases (RTK), like hepatocyte growth factor receptor (HGFR/MET), the human epidermal growth factor receptor 4 (HER4) or the insulin like growth factor 1 receptor (IGF1R) at the cell surface^38^. If fewer RTKs are proteolytically cleaved, they accumulate at the cell surface and activate alternative signaling pathways that induce proliferation and contribute to tumor progression^38^. Upstream of these signaling pathways is the matrix metalloprotease ADAM17, cleaving a variety of different substrates, of which at least six substrates bind to growth factors, such as the EGFR^39,40^. By activating EGFR signaling, but also by shedding RTKs on the cell surface, ADAM17 plays a crucial role in mediating intracellular signal transduction and promotes tumor proliferation, migration and metastasis^41–43^. In both hepatocellular carcinoma and ovarian cancer, treatment with cisplatin leads to ADAM17-dependent release of cleaved growth factors and subsequent activation of the EGFR/PI3K/AKT pathway. Inhibition of ADAM17 resulted in sensitivity to cisplatin treatment and induced apoptosis^27,44^. In light of these findings, our central hypothesis is that olaparib treatment increases activation of ADAM17, thereby downregulating olaparib-induced apoptosis. Accordingly, combined inhibition of ADAM17 and PARP leads to sensitivity in the treatment of ovarian cancer.

Already in two 2D culture experiments we could show that the combination of olaparib and the ADAM17 inhibitor GW possesses strong synergistic effects. Interestingly, the combinatorial effect extends beyond HRD status, as the HRD negative cell line IGROV-1 also demonstrates response. The reason for this, could be the numerous mutations in the risk genes, which have a significant impact on homologous recombinational repair. This is in line with the results of Konstantinopoulos et al. who found in a clinical phase Ib trail a comparable therapeutic response in both the HR proficient and HR deficient subgroup treated with olaparib and alpelisib (PI3K inhibitor). Since 61% of patients did not have a *BRCA* germline mutation and 93% of patients were cisplatin resistant, it was hypothesized that alpelisib inhibits HR and re-sensitizes ovarian cancers with acquired HR proficiency to olaparib therapy^45^.

To emphasize the translational aspect of our work, we performed corresponding experiments on primary cells. Interestingly, already the inhibition of ADAM17 leads to a strong reduction in viability. As described earlier, ADAM17 activity is divided into constitutive catalytic and induced shedding activity^46^. Sahin et al. observed that ADAM17 cleaves and releases its substrates epigen from the cell surface of fibroblasts^47,48^. However, it was possible to strongly upregulate this basal activity by treating the tumor cells with the phosphatase inhibitor pervanadate, the phorbol ester PMA or the calcium ionophore ionomycin. Indeed we and others demonstrated that cisplatin treatment leads to upregulation of ADAM17 and increased release of growth factors that mediate cisplatin resistance in ovarian cancer and hepatocellular carcinoma, respectively^26,44^. At the same time, blockade of ADAM17 leads to downregulation of constitutive and induced shedding activity. This contributes to reduced growth factor release and tumor proliferation. The downregulation of constitutive shedding, can be well observed in the 2D cell culture experiments of primary cells, as they are initially very sensitive to treatment with the ADAM17 inhibitor GW. With the addition of olaparib to ADAM17 inhibition, apoptosis is induced and a strong increase in caspase activity is observed.

Moreover, our study shows that the olaparib effect was significantly enhanced by ADAM17 inhibition, provided that olaparib exerted an initial cytotoxic effect. Therefore, we hypothesize that ADAM17 is enhanced by olaparib-induced apoptosis. It has previously been shown that apoptosis induced by DNA damage increases the activity of ADAM17 and upregulates its substrate shedding on cell membrane^49–52^. Enhanced caspase activity leads to loss of membrane integrity and an increase in cytosolic Ca^2+^. As a consequence it comes to an externalization of phosphatidylserine (PS) and directs ADAM17 to the cell surface^53^. Activated by cell death, ADAM17 forces the cleavage of membrane-bound growth factors such as IL-6 or TNF alpha. Hereby, RTKs and their downstream signaling, such as the EGFR/PI3K/AKT pathway are activated, which enable suppression of apoptosis and provide the tumor with resistance to therapeutic treatment^44^. In contrast, blocking ADAM17 disrupts the antiapoptotic process and enhances the response to olaparib treatment. This strengthens the hypothesis that for combinational treatment an initial trigger for apoptosis is needed to provoke a synergistic effect.

This is the first study to investigate the function of ADAM17 in mediating resistance to olaparib treatment. The results suggest that ADAM17 is a promising target in overcoming resistance mechanisms in ovarian cancer and is worth investigating in further studies. Furthermore, the translation of our findings into a 3D spheroid model with primary tumor cells makes a significant contribution to reflect the in vivo conditions, highlighting the value of our work.

## Materials and methods

### Cell culture and isolation of primary cells

Adherent ovarian cancer cell lines OVCAR-8 (RRID: CVCL_1629*)*, IGROV-1 (RRID: CVCL_1304) and SKOV-3 (RRID: CVCL_0532), purchased from American Type Culture Collection (ATCC) were grown with RPMI-1640 medium containing 10% fetal bovine serum (Gibco Life Technologies), 1% L-Glutamine (Sigma-Aldrich, St. Louis, MO, USA) and penicillin-streptomycin (3000 U pen./30,000 µg str. per 500 mL RPMI-1640; Biochrom). The cell culture flasks were maintained in an appropriate incubator (Forma Scientific; Thermo Scientific, Pinneberg) at 37 °C with 5% CO_2_ and 70–80% humidity for the duration of cultivation. Subcultivation was performed based on respective growth rates when confluency reached 70–80%. As a routine procedure, potential contamination with mycoplasma was ruled out using MycoAlert TM kits (Lonza, #LT07-118). Primary tumor cells of 5 ovarian cancer patients (see Table 4 for clinical characteristics) were obtained through surgical extraction of tumor tissue in ovarian cancer patients that presented at an advanced stage. Cells were then isolated from the tumor tissue and cultivated for expansion as described above. Autenticity of cell lines and primary cells was determined by analysis of the STR marker profile as previously described^54^.

## TruRisk genepanel sequencing

To conduct TruRisk Genepanel sequencing, we extracted DNA from cryopreserved tumor tissue, blood and from harvested cells. For extraction and purification, we used the AllPrep DNA-RNA-miRNA Universal Kit from Qiagen (Qiagen, #80224). DNA enrichment was performed using Illumina DNA Prep protocol (Illumina, #20025523 and #20025524). Subsequent DNA sequencing was performed by the TruRisk Genepanel assembled by the German Consortium for hereditary Breast and Ovarian Cancer considering the risk genes *ATM*,* BRCA1*,* BRCA2*,* BRIP1*,* CDH1*,* CHEK2*,* EPCAM*,* MLH1*,* MSH2*,* MSH6*,* PALB2*,* PMS2*,* PTEN*,* RAD51C*,* RAD51D*,* STK11*,* TP53.*

### Homologous recombination deficiency score

The individual HRD score was determined for the cell lines as well as for the primary cell UF-352 by Array-based Comparative Genomic Hybridization (aCGH). For this, 0.5–1 µg tumor DNA and an equivalent amount of reference DNA (Agilent female) was utilized. A 2 × 400k + SNP array (Agilent Technologies Inc., Santa Clara, Ca. design: 028081) was chosen for the primary cells. For cell lines, we used 4 × 180k CGH + SNP arrays, which have high resolution for *BRCA1*,* BRCA2* and *TP53* genes (86822_V3_HRD_4 × 180k_CGH_SNP_Kiel1). According to the Array-Based CGH for Genomic DNA Analysis, version 7.4, hybridization and purification were performed. Subsequently, the fluorescence of the labeled DNA was detected by SureScan Dx microarray scanner G5761A (Agilent Technologies Inc., Santa Clara, Ca.). Agilent Feature Extraction software 3.0.5.1 (Agilent Technologies Inc., Santa Clara, Ca.) was selected to read the raw data and Agilent Cytogenomics 3.0.6.6 (Agilent Technologies Inc., Santa Clara, Ca.) evaluation software was used to analyze the data. The HRD score was calculated according to the criteria of Timms et al.^55^. In the HRD score, telomeric imblances (TAI) greater than 11 Mb, large scale transitions (LST) greater than 10 Mb, and loss of heterozygosity (LOH) greater than 15 Mb are considered. Summation of the individual events at LOH, TAI and LST results in the calculated HRD score.

## Workflow of 2D drug treatment and the subsequent readout procedure

**Fig. 5:**
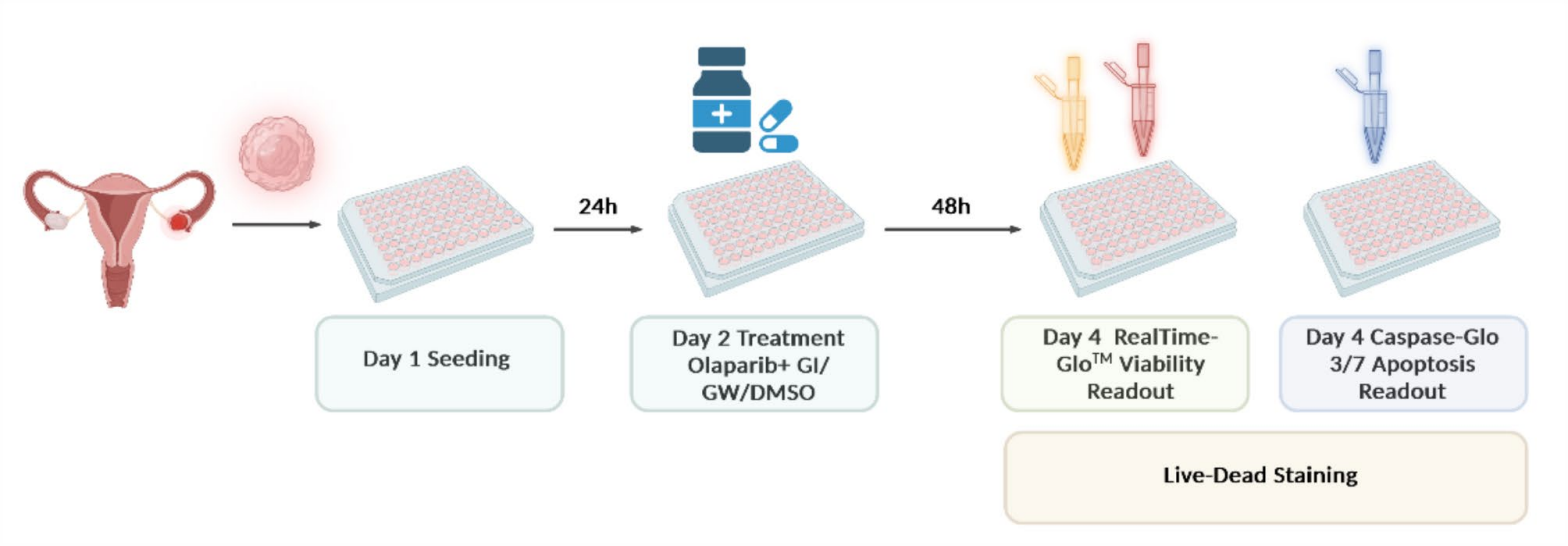
Workflow of 2D drug treatment and subsequent readout procedure: 2D multiplex assay workflow for testing combinatorial effects of GI / GW / DMSO and Olaparib: Cells are seeded on day 1 in 96 well plates and treated on day 2 with the combination of inhibitors GI / GW or control DMSO and increasing concentrations of olaparib. After 48 h incubation, viability and apoptosis will be determined using the endpoint multiplex readout assay ApoLive-Glo. Alternatively, live-dead staining was performed. Image was created with BioRender.com.

The ovarian cancer cells were seeded in different amounts per well (OVCAR-8, SKOV-3, UF-357, UF-364, UF-477, UF-510: 5 × 10^3^; UF-352: 6 × 10^3^; IGROV-1: 10 × 10^3^) into Brand 96-well plates (BRAND #781970, Wertheim, Germany) and were incubated for 24 h Fig. 5). Following, the medium was refreshed, and DMSO as a solvent control, the ADAM10 inhibitor GI254023X (Aobious, #3611; 3 µM) and the ADAM10/ADAM17 inhibitor GW280264X (Aobious, #3632; 3 µM) were applied to the plate in triplicates. We used a constant concentration GI/ GW /DMSO of 3 µM, since the strongest inhibitory effect with simultaneously low off-target effect is described here^49,56,57^. After another incubation of two hours, PARPi olaparib (AZD 2281, Axon Medchem, diluted in PBS with 0.05% DMSO) was added at increasing levels of concentration, ranging from 0.1 to 200 µM. Read-out was performed 72 h after treatment. The Multiplex Assay ApoLive-Glo System (Promega #G6411, Fitchburg, USA), which measures cell viability and caspase 3/7 activity, was used as indicated in the manufacturer’s instructions (TM325) to quantify the therapeutic efficacy of the applied treatment. A microplate-reader (Infinite Spark, Tecan, Männedorf, Switzerland) was used to measure both cell viability and caspase 3/7 activity. Viable cells were detected as relative fluorescence units (RFU) using 400 Ex/505 Em excitation and emission filters, respectively. Apoptotic cells were measured in relative luminescence units (RLU) based on caspase 3/7 cleavage using the Caspase-Glo 3/7 Assay. As an alternative method to demonstrate the effects of the combination treatment, the cells were stained 72 h after treatment. Dye was applied to the wells to stain living cells with 0,1 µg/ml of Calcein-AM (BioLegend, #425201) and dead cells with 5 µg/mL of propidium iodide (PI) (BioLegend, #421301). As a nuclear counterstain Hoechst33342 (Invitrogen, #H1399) was used in a final concentration of 1 µg/mL. At room temperature, the plates were incubated and then placed into the NYONE Scientific (SYNENTEC) to be imaged using the 10× objective and the following settings: brightfield: Ex: BF; Em: Green (530/43 nm); PI: Ex: Lime (562/40 nm); Em: Red (628/32 nm), Hoechst33342: Ex: UV (377/50 nm), Em: Blue (452/45 nm). Representative images are shown, and a scale bar was added while scaling the images in GIMP 2.10.14 (GNU Image Manipulation Program).

### Workflow of 3D drug treatment and subsequent readout procedure

**Fig. 6:**
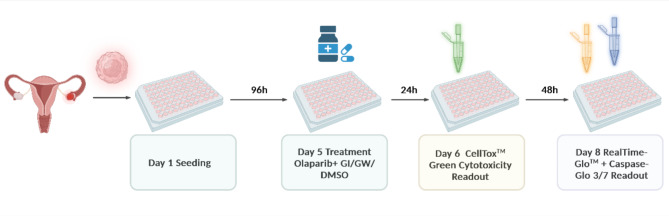
Workflow of 3D drug treatment and the subsequent readout procedure: 3D multiplex assay workflow for testing combinatorial effects of GI/ GW /DMSO and Olaparib: cultured cells are seeded on day 1 in 96-well plates and treated on day 5 with the combination of inhibitors GI/GW or control DMSO and increasing concentrations of Olaparib. After 24 h incubation, CellTox Green Cytotoxicity Assay and on day 8 RealTime-Glo MT Cell Viability Assay was performed. Image was created with BioRender.com.

For better applicability of the results to 3D tumor tissue, the experiments were also conducted on cells that had formed into spheroids, in addition to cells in monolayer. To achieve the formation of spheroids, ovarian cancer cells were seeded in ultra-low attachment plates (Corning #7007, New York, USA). The optimal cell counts were previously assessed and were as follows: 2 × 10^3^ (OVCAR-8), 3 × 10^3^ (UF-357, UF-364, UF-510). Daily images of spheroid formation and measurements of spheroid diameters were taken at 4x and 10x magnification with either an automated imaging system consisting of a CELLAVISTA 4K imager SYBOT-1000 & Cytomat 2 C-LiN (SYNENTEC GmbH) or the NYONE Scientific (SYNENTEC). After an incubation period of 48 h, medium was carefully withdrawn from the wells and replaced of fresh medium. Subsequently the solvent DMSO and inhibitors, as well as the treatment with PARPi Olaparib were applied in the same manner as stated before, leaving an incubation period of two hours in between those steps. The next day, a final dilution of 1:2000 of the CellTox Green cytotoxicity (Promega #G8743) dye was added. Three hours later the spheroids were imaged with the same automated imaging systems as above using the following settings: brightfield (Ex: BF, Em: Green (530/43 nm)), CellTox Green (Ex: Blue (475/28 nm); Em: Green (530/43 nm)). Imaging was repeated every 24 hours. After the final measurement 72 h following treatment, two separate luminous assays were used to determine endpoint viability and caspase3/7. Next, the number of viable cells was determined by applying the RealTime-Glo MT Assay (Promega, #G9712) and the results were measured using Tecan Spark after 1 h of incubation. Proceeding with the application of the Caspase-Glo 3/7 Assay System (Promega, #G8093) at room temperature, measurements were then performed after 1 h of incubation (Fig. 6).

### Statistical analyses

The means from three biological replicates with time-shifted cultivation and treatment were reported for both the OVCAR-8, IGROV-1 and SKOV-3 cell lines used in the 2D experiments as well as the OVCAR-8 used in the 3D experiments. Based on a passage number restriction, we performed three biological replicates in one time frame for the primary cell experiments. The IC_50_ and EC_50_ of the cell lines were determined for each treatment (GI /GW /DMSO) and readout (viability, caspase) by fitting standard four-parameter logistic regression dose-response curves using GraphPad-Prism version 10 (GraphPad Softwares, Boston, MA). To quantify the effect of combination therapy relative to mono-therapy in cell lines, the dose reduction index (DRI_50_) was calculated assuming a common maximum (top), minimum (bottom) and slope parameter from each pair of fitted dose-response curves (olaparib + GI /GW /DMSO). The DRI_50_ represents the magnitude of dose reduction required to achieve IC_50_ compared to mono-therapy with olaparib. The DRI_50_ allows to distinguish between antagonistic (DRI_50_ > 1), additive (DRI_50_ = 1) and synergistic effects (DRI_50_ < 1) of the combinatorial treatment. The primary cells exhibited such a different overall shape of the curves (olaparib + GI /GW /DMSO), that only separate curve with unconstrained parameters were estimated for each treatment condition and no DRI_50_ could be estimated. Normal distribution was checked by the Shapiro-Wilk test. To compare the means of more than two groups (readouts at different concentrations), a one-way Anova was performed with a Dunnett’s multiple comparison. To determine the response of the combination treatment, two-way Anova followed by Turky’s multiple comparison test was performed. Statistical significance was assumed at a p-value < 0.05.

### Ethics statement

The study was conducted in accordance with the Declaration of Helskinki. Approval was granted by the Institutional Review Board of the University Medical Center Schleswig-Holstein, Campus Kiel (AZ: D563/21). Written informed consent was obtained from all patients.

## Conclusions

This study investigated the function of ADAM17 in mediating resistance to olaparib treatment in ovarian cancer. The combination with the ADAM17 inhibitor GW significantly enhanced the apoptotic effect of olaparib, suggesting a strong synergistic effect. This renders ADAM17 a valuable alternative target in overcoming resistance mechanisms. The translational approach of this study allowed us to validate the initial results in a more complex 3D spheroid model with primary tumor cells. Nevertheless, further studies are needed to elucidate the function of ADAM17 in mediating PARPi resistance mechanisms in ovarian cancer therapy, in order to identify suitable therapy formats including combinatorial treatments to overcome PARPi resistance in this devastating disease.

## Data Availability

Provided there were no ethical or data protection restrictions according to the European General Data Protection Regulation (GDPR), the datasets analyzed in the current study were made available in Kiel University’s research data repository ‘opendata@uni-kiel’ (https://doi.org/10.57892/100-80).

## Abbreviations

PARPi: Poly (ADP-ribose) polymerase inhibitors
HRD: Homologous recombination deficiency
HGSOC: High-grade serous ovarian cancer
BRCA1/2: Breast cancer gene 1/2
PFS: Progression free survival
ADAM17: A disintegrin and metalloproteases 17
EGFR: Epidermal-growth-factor-receptor
GI: GI254023X
GW: GW280264X
IC50: Half-maximal inhibitory concentration
EC50: Half-maximal effective concentration
DRI50: Dose reduction index
RFU: Relative flourecence units
RLU: Relative luminescence units
PI: Propidium iodide
HR: Homologous recombination
RTK: Receptor tyrosine kinases
IGF1R: Insulin like growth factor 1 receptor
TAI: Telomeric imblances
LST: Large scale transitions
LOH: Loss of heterozygosity
STR: Short tandem repeats
